# Development and validation of a clinical prediction rule for development of diabetic foot ulceration: an analysis of data from five cohort studies

**DOI:** 10.1136/bmjdrc-2021-002150

**Published:** 2021-05-25

**Authors:** Francesca M Chappell, Fay Crawford, Margaret Horne, Graham P Leese, Angela Martin, David Weller, Andrew J M Boulton, Caroline Abbott, Matilde Monteiro-Soares, Aristidis Veves, Richard D Riley

**Affiliations:** 1 Centre for Clinical Brain Sciences, University of Edinburgh, Edinburgh, UK; 2 The School of Medicine, University of St Andrews, St Andrews, Fife, UK; 3 Research, Development and Innovation, NHS Fife, Dunfermline, Fife, UK; 4 Centre for Population Health Sciences, University of Edinburgh, Edinburgh, UK; 5 Ninewells Hospital, NHS Tayside, Dundee, UK; 6 Victoria Hospital, NHS Fife, Kirkcaldy, UK; 7 Usher Institute, University of Edinburgh, Edinburgh, UK; 8 Division of Diabetes, Endocrinology and Gastroenterology, University of Manchester & Manchester Royal Infirmary, Manchester, UK; 9 University of Miami, Miami, Florida, USA; 10 Manchester Metropolitan University, Manchester, Greater Manchester, UK; 11 MEDICIDS, CINTESIS, Faculty of Medicine, University of Porto, Porto, Portugal; 12 Beth Israel Deaconess Medical Center, Harvard Medical School, Boston, Massachusetts, USA; 13 School of Primary, Community and Social Care, Keele University, Keele, UK

**Keywords:** prevention, foot ulceration, epidemiology

## Abstract

**Introduction:**

The aim of the study was to develop and validate a clinical prediction rule (CPR) for foot ulceration in people with diabetes.

**Research design and methods:**

Development of a CPR using individual participant data from four international cohort studies identified by systematic review, with validation in a fifth study. Development cohorts were from primary and secondary care foot clinics in Europe and the USA (n=8255, adults over 18 years old, with diabetes, ulcer free at recruitment). Using data from monofilament testing, presence/absence of pulses, and participant history of previous ulcer and/or amputation, we developed a simple CPR to predict who will develop a foot ulcer within 2 years of initial assessment and validated it in a fifth study (n=3324). The CPR’s performance was assessed with C-statistics, calibration slopes, calibration-in-the-large, and a net benefit analysis.

**Results:**

CPR scores of 0, 1, 2, 3, and 4 had a risk of ulcer within 2 years of 2.4% (95% CI 1.5% to 3.9%), 6.0% (95% CI 3.5% to 9.5%), 14.0% (95% CI 8.5% to 21.3%), 29.2% (95% CI 19.2% to 41.0%), and 51.1% (95% CI 37.9% to 64.1%), respectively. In the validation dataset, calibration-in-the-large was −0.374 (95% CI −0.561 to −0.187) and calibration slope 1.139 (95% CI 0.994 to 1.283). The C-statistic was 0.829 (95% CI 0.790 to 0.868). The net benefit analysis suggested that people with a CPR score of 1 or more (risk of ulceration 6.0% or more) should be referred for treatment.

**Conclusion:**

The clinical prediction rule is simple, using routinely obtained data, and could help prevent foot ulcers by redirecting care to patients with scores of 1 or above. It has been validated in a community setting, and requires further validation in secondary care settings.

**Significance of this study**

**What is already known about this subject?**

- Foot ulcers can lead to amputation and are costly to healthcare providers.
- Prediction of who will develop an ulcer means preventative therapies can be targeted appropriately.
- Current prediction models do not give precise risk estimates but a descriptive term instead, for example, ‘intermediate risk’, which is hard to interpret.

**What are the new findings?**

- A simple, validated clinical prediction rule that quantifies risk with scores 0 to 4 using the sum of:
  Score 1 if insensitive to a 10 g monofilament.
  Score 1 if any pedal pulse is absent.
  Score 2 if there is history of previous ulcer or amputation.
- Risks of developing an ulcer for each score are:
  Score 0—risk is 2.4% (95% CI 1.4% to 3.9%).
  Score 1—risk is 6.0% (95% CI 3.5% to 9.5%).
  Score 2—risk is 14% (95% CI 8.5% to 21%).
  Score 3—risk is 29% (95% CI 19% to 41%).
  Score 4—risk is 51% (95% CI 38% to 64%).
- Patients with a score of 1 or more could benefit from preventative treatment.

**How might these results change the focus of research or clinical practice?**

- Health professionals will be able to quickly and easily estimate risk of foot ulceration and so direct preventative therapies at those most likely to benefit.

## Introduction

Diabetes-related foot ulcers have severe consequences for the individual and healthcare systems providing foot care.^1^ Some ulcers lead to lower extremity amputation and ulcers have been linked with higher rates of mortality.^2^ Moreover, diabetes-related amputations are now increasing in young and middle-aged adults.^3^ In 2016, there were 130 000 people with diabetes discharged from a US hospital with a lower extremity amputation.^4^ Estimated costs of treating ulcers vary from $10 000 to $35 000 per ulcer,^5^ and the annual direct costs in the USA alone of $176 billion, of which up to a third are related to the lower extremity.^6^ Similar costs per capita are seen globally,^7 8^ and the National Health Service in England could save £250 million per year if the prevalence of foot ulceration was reduced by one third.^1^

Since the early 1990s, researchers have been developing tools to predict the risk of a diabetes-related foot ulceration or lower extremity amputation. These tools vary in complexity and include a range of patient data.^9^ For example, the QDiabetes tool will calculate risk of amputation or blindness within the next 10 years, though it does not give risk of foot ulceration. Seven predictive tools for foot ulcer (including the American Diabetes Association, the University of Texas Foot Risk System, and the International Working Group on the Diabetic Foot risk classification systems) were tested in a population of 446 people in Portugal. The models all demonstrated relatively high levels of accuracy with C-statistics between 0.75 and 0.86. However, the number of elements required by each predictive model varied from 4 to 15. This variation is reflected in current diabetes clinical guidelines, which recommend the use of between 8 and 10 individual elements from the patients’ history or test results, or different combinations of the same, for risk assessment.^10–12^ None of these predictive models gives a quantified risk, but a descriptive term such as ‘high risk’ or a recommendation such as ‘refer to foot clinic’.

Certain interventions have been shown to reduce the incidence of foot ulcers. A recent systematic review and meta-analysis found some evidence of effective interventions to prevent foot ulceration. Meta-analyses of dermal infrared thermometry (relative risk 0.41 (95% CI 0.19, 0.86)), complex interventions (relative risk 0.59 (95% CI 0.38, 0.90), and custom-made footwear and offloading insoles (relative risk 0.53 (95% CI 0.33, 0.85)) all reduced the incidence of foot ulcers.^13^ Given this existing knowledge of predisposing factors for foot ulceration and the availability of preventative interventions, our aim was to develop and validate a prognostic model and subsequent clinical prediction rule (CPR) to provide a risk estimate for an individual using his or her own data. The CPR should be accurate, simple to use, inexpensive, and produce a quantified risk of foot ulceration within a meaningful timeframe, for patients in primary and secondary care settings.

## Research design and methods

### Source of data

This PODUS 2020 (Prediction of Diabetic foot Ulcerations) project used individual participant data (IPD) from the PODUS 2015 project, which identified predictors of foot ulceration in diabetes by systematic review and meta-analysis (see the online supplemental material for details of PODUS 2015).^14^ The search strategies for Medline and Embase were rerun to find new studies published since 2015 and searched to May 2017. We identified one eligible study but a request for data was unsuccessful.^15^ The PODUS 2020 inclusion criteria were:

10.1136/bmjdrc-2021-002150.supp1 Supplementary data

- Studies that recruited people 18 years old or older with a diagnosis of diabetes.
- Participants were ulcer-free at time of recruitment (or if the study recruited some individuals with a current ulcer, it was possible to remove those individuals from the analysis), participants with a history of previous ulcer were eligible.
- Predictors of foot ulcer were assessed at baseline.
- Foot ulcer presence/absence was ascertained at follow-up.

Data from four cohort studies were used to develop the prognostic model and subsequent CPR.^16–19^ Data from a fifth cohort study, only available remotely via a Safe Haven facility, were used for external validation.^10 20^ The validation dataset was an electronic register, which had taken data from General Practice records and Information Services Division NHS Scotland. The other PODUS 2015 studies were either no longer available^21^ or did not have the required predictors.^22–25^

All studies were assessed for their risk of bias using the Prediction model Risk of Bias Assessment Tool (PROBAST).^26^ Recruitment dates ranged from May 1995 to November 2007 in the development datasets, and the last follow-up date was December 2008. In the validation dataset, recruitment dates ranged from January 2001 to December 2006 and the final follow-up date was 2007. These studies are described extensively elsewhere.^14^

### Participants

Of the four studies used for model development, two studies collected data from people who received care in UK community settings^16 17^ and two collected data from people in hospital foot clinics set in mainland Europe and the USA.^18 19^ The validation dataset had data from a UK electronic health register. Participants met the above criteria and received standard care for the setting.

### Outcome

The outcome was any definition of foot ulceration as used by the contributing studies occurring within 2 years of baseline, as assessed by podiatrists or self-report questionnaires. The largest contributing study,^16^ with 6478 participants comprising 78% of the total model development dataset, had data on whether an ulcer had developed within 2 years from baseline, but not the precise date of ulceration. The other development and validation studies either gave time to ulceration or date of last follow-up, so that their data could be harmonized with the largest dataset.^16^

In three of the development datasets,^16 17 19^ the assessment of outcome was blinded to predictors where possible. One of the predictors included amputation, which cannot be hidden from the assessor of ulcer outcome.

### Selection of predictors

We chose three binary predictors for inclusion in prognostic model and subsequent CPR based on their clinical plausibility, availability, and testing in PODUS 2015 (see the online supplemental material).^14^ The three predictors were (a) insensitivity to a 10 g monofilament, (b) an absent pedal pulse in either foot, and (c) previous history of ulceration or amputation.

Healthcare professionals carried out the test of touch sensation using a 10 g monofilament by applying the monofilament to the plantar aspect of the foot at a variety of sites. Participants then confirmed whether they felt the monofilament.

Healthcare professionals palpated two pulses in each foot, the dorsalis pedis and the posterior tibial pulses. However, a minority may be missing the dorsalis pedis pulse and be healthy.^27^

Previous history of ulceration or amputation was ascertained either at initial assessment or from patient records. Patients were test-positive for previous history if either a foot ulcer or a lower extremity amputation had occurred prior to baseline data collection.

### Statistical analyses methods—handling of predictors

The studies varied in the data recorded for insensitivity to 10 g monofilaments and absent pedal pulses. For monofilaments, the number and place of sites on the foot tested by monofilament varied between studies. For pulses, some studies gave the total number of pulses per person, and others had recorded the absence/presence of the two individual pulses on each foot. However, for consistency across studies, the following coding was adopted:

- Insensitivity to monofilaments was coded as 1 if the participant was insensate anywhere on the foot and 0 if the participant could feel the monofilament at all sites.
- Absent pulses was coded as 1 if any of the four pulses (two on each foot) were missing and 0 if all four were present.

Previous history (ulcer and amputation) was consistently coded in the different studies. Data on previous foot ulceration and previous amputation were combined because of the association of amputation with ulceration—they both suggest a propensity to ulcerate.^28^ For the prognostic model analysis, previous history was coded as 1 if the participant had a previous ulcer or amputation, and 0 if the participant had never had an ulcer or amputation.

Summary statistics were calculated for all the predictors and an extensive description of all the datasets can be found elsewhere.^14^

### Statistical analyses methods—underlying statistical model

The prediction model was logistic regression with the binary outcome of ulcer by 2 years. The predictors were monofilaments, pulses, and previous history of ulcer or amputation, which were forced into the model regardless of statistical significance. We checked with shrinkage factors that the size of the dataset and number of outcomes were adequate to fit the model. See the online supplemental material for details.

### Statistical analyses methods—conversion of the prognostic model to a CPR

A general method for converting a prognostic model to a CPR is described by Steyerberg.^29^ In essence, the coefficients of the prediction model are used to generate a scoring system (see the online supplemental material), by rounding coefficients and creating risk groups.

### Statistical analyses methods—validation of predictive performance

For each participant in the validation dataset,^10 20^ we calculated the CPR score from the individual’s results for monofilaments, pulses, and previous history.

For each score, the actual risk of ulcer in the validation dataset was compared with the predicted risk of ulcer. Discrimination was assessed by calculating the C-statistic and visual examination of a receiver operating characteristic (ROC) plot. A perfectly discriminating CPR would have a C-statistic of 1, while a CPR with no discrimination beyond chance would have a C-statistic of 0.5. Calibration was assessed with calibration-in-the-large, calibration slope, and calibration plots.^29^ A perfectly calibrated CPR would have a calibration slope of 1 and a calibration-in-the-large of 0.

The CPR is a simplification of the prognostic model and so may result in a loss of information. Hence, the performance of the prognostic model was compared with the CPR score in the validation dataset.

### Statistical analyses methods—net benefit and decision curve

Finally, we conducted a net benefit analysis to investigate how useful the CPR could be in practice.^30^ The net benefit analysis compared a ‘treat all’ and ‘treat none’ strategy to ‘treat some’ where who does and does not receive treatment is decided by the CPR score. The net benefit analysis indicated whether the CPR could have a clinical impact and was assessed with decision curves.^30^

### Reporting and software

This study adhered to the Transparent Reporting of a multivariable prediction model for Individual Prognosis or Diagnosis (TRIPOD) reporting guidelines and include a TRIPOD checklist in the online supplemental material.^31^ Software used were SAS V.9.4 (www.sas.com) and R V.3.4.2 (https://cran.r-project.org/) for all analyses. The pROC,^32^ meta,^33^ and rms^34^ packages in R were used.

## Results

Results of the critical appraisal with the PROBAST tool are given in the online supplemental material. Overall, studies were considered to be at a low risk of bias and applicable to the research purpose. In one development dataset and the validation dataset, outcome was assessed with knowledge of the predictors.^10 18 20^

### Participants

Participant flow diagrams for four studies are given in the online supplemental material. These show how many participants did and did not have complete data. One study did not need a flow diagram as it had complete data for the outcome and all three predictors.^18^ The number of patients in the development datasets was 8404, and the number who contributed to the analyses was 8255 (98%). The percentage with complete data in the validation dataset was 3324 (97.4%), again high enough not to require multiple imputation.

The median age of the participants was older in the validation dataset (67 years) versus development (64 years) datasets. The percentage of men ranged from 45.6% to 56.6% in the five datasets (see table 1).

**Table 1:** Demographic results for the development studies (Abbott, Crawford, Monteiro-Soares, Pham)^16–19^ and the validation study (Leese)^10 20^

Study	Age (years) mean±SD	Duration of diabetes (years) mean±SD	Men, N (%)
Abbott	61.3±13.2	8.2±8.2	3515 (53.2)
Crawford	70.5±10.0	8.8±8.4	611 (51.2)
Monteiro-Soares	64.3±10.4	15.8±10.4	164 (45.6)
Pham	58.3±12.5	13.9±10.8	124 (50.0)
All development	62.7±13.1	8.8±8.6	4414 (52.5)
Leese	65.1±13.1	6.8±7.8	1931 (56.6)

The community-based studies^16 17^ had smaller percentages of people insensitive to monofilaments or with previous history compared with the secondary care-based studies,^18 19^ but broadly comparable percentages with absent pulses (see table 2). The risk of ulcer in the community-based studies^16 17^ was at least 10% lower than the secondary based care based studies. The results from the validation dataset^10 20^ were more similar to the results from the community based studies^16 17^ than the secondary care based studies.^18 19^

**Table 2:** Predictor and outcome data in the development studies (Abbott, Crawford, Monteiro-Soares, Pham)^16–19^ and validation study (Leese)^10 20^

Study	N in study	% (n) insensitive to monofilament	% (n) missing at least one foot pulse	% (n) with previous ulcer or amputation	% (n) with ulcer outcome at 2 years	% (n) with complete data
Abbott	6603	19.4 (1278)	29.6 (1957)	4.7 (312)	4.4 (291)	98.1 (6478)
Crawford	1193	22.3 (266)	18.8 (224)	7.2 (86)	1.9 (23)	98.5 (1175)
Monteiro-Soares	360	46.1 (166)	20.3 (73)	38.1 (137)	14.4 (52)	100 (360)
Pham	248	74.6 (185)	14.5 (36)	71.4 (177)	27.8 (69)	97.6 (242)
All development studies	8404	22.5 (1895)	27.2 (2290)	8.5 (712)	5.2 (435)	98.2 (8255)
Leese	3412	20.7 (707)	14.0 (478)	5.7 (196)	3.9 (133)	97.4 (3324)

### Prognostic model development

The number of patients from the development datasets used in the logistic regression models was 8255, with 430 people developing ulcers and 7825 people remaining ulcer-free.

On the log-odds scale, for the logistic regression model using the three clinical predictors, the coefficient for monofilaments was 1.11, for pulses 0.70, and previous history 1.95. These correspond to ORs of 3.00 (95% CI 2.39 to 3.76), 2.01 (95% CI 1.62 to 2.51), and 7.02 (95% CI 5.40 to 9.13), respectively. The estimate of baseline risk was 2.2% (95% 1.7% to 2.8%). See the online supplemental material for the underlying logistic regression equation that forms the prognostic model.

### CPR development

After examination of the prognostic model coefficients and the corresponding risks of developing an ulcer given in the online supplemental material, a CPR was created based on the following scoring system:

- Score 1 if insensitive to a 10 g monofilament.
- Score 1 if any pedal pulse is absent.
- Score 2 if there is history of previous ulcer or amputation.

This CPR therefore gives scores from zero to four. The modeling procedure was repeated (see the online supplemental material) with CPR score as the only predictor. Baseline risk was 2.4% (95% CI 1.7% to 3.4%), and the OR for CPR score was 2.57 (95% CI 2.36 to 2.81). Risk of ulceration for each score is given in table 3.

**Table 3:** Population-based probability of ulcer at 2 years for each CPR score, calculated using Pavlou’s method for population-average estimates

CPR score	N patients	Probability of ulcer at 2 years	95% CI
0	4646	0.024	(0.014 to 0.039)
1	2406	0.060	(0.035 to 0.095)
2	676	0.140	(0.085 to 0.213)
3	358	0.292	(0.192 to 0.410)
4	169	0.511	(0.379 to 0.641)

CPR, clinical prediction rule.

Shrinkage factors for both the prognostic model and the CPR were very close to 1, showing that the sample size was adequate for the analyses (see the online supplemental material for details).

### Validation of the CPR in the validation dataset

**Figure 1:**
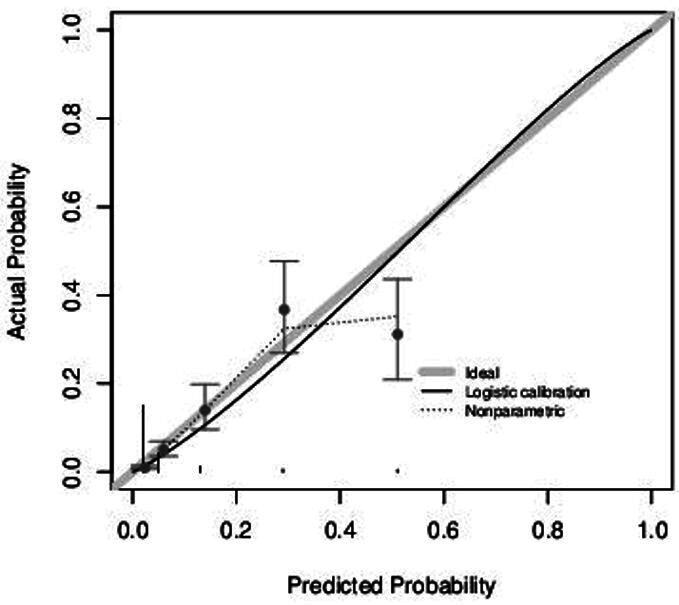
Calibration plot from the external validation of the CPR. Gray line indicates perfect calibration; black curve is the estimated calibration curve. The five groups correspond to CPR scores of 0–4, and vertical lines show the width of the 95% CI. CPR, clinical prediction rule.

In the validation dataset, there were 3324 participants with suitable data, of whom 128 had an ulcer by 2 years and 3196 remained ulcer-free.^10 20^ The validation plot suggests excellent calibration of risks in the lower risk groups, but slight miscalibration at higher risk groups (see figure 1); however, the net benefit analysis below would recommend the same clinical pathway despite any miscalibration. The calibration slope was 1.139 (95% CI 0.994 to 1.283) and calibration-in-the-large was −0.374 (95% CI −0.561 to −0.187). See the online supplemental material for calibration results in the prognostic model, which are very similar to those of the CPR.

Examination of the ROC curve (see the online supplemental material) suggests very little loss of discrimination performance when using the CPR compared with the prognostic model. The area under the ROC curve for the CPR was 0.829 (95% CI 0.790 to 0.868) and for the prognostic model was 0.834 (95% CI 0.794 to 0.873).

### Potential impact of the CPR

The net benefit analysis aimed to balance the risk of not offering treatment to people who would develop an ulcer against treating people who would not develop an ulcer. If the overall risk of ulceration is very high, there will be a benefit to treating everyone. If the overall risk of ulceration is very low, it may not be worth offering treatment to anyone. Between these two extremes, the net benefit analysis suggests that offering treatment to those people with a risk of 6% or more, that is, a CPR score of 1 or more, would correctly weigh risk of ulceration against unnecessary treatment (see online supplemental material).

## Conclusion

A CPR was developed and validated to predict foot ulceration within 2 years in people with diabetes. The data came from a large international dataset, which was assembled using a systematic review and IPD meta-analyses.^14 35^ The three predictors that form the CPR are easy to collect during patient foot examinations, are usually recorded in health records and therefore this CPR can be applied at the point of care. The 10 g monofilament test is inexpensive and widely used in foot risk assessments (eg, https://gpnotebook.com/simplepage.cfm?ID=x2020063010498191128), and the assessment of pedal pulses is a standard part of routine foot care. A history of lower extremity amputation or foot ulceration is both well-known risk factors and is highly likely to have been recorded in patient’s health records across different healthcare settings.

Other advantages of this CPR are that it requires very little calculation by the end-user and quantifies a person’s risk of foot ulceration over a 2-year timescale in a way that is easy to understand. Simplicity, ease-of-use, and cost are all important factors that can affect whether or not a CPR is used in clinical practice. This is especially true in the context of the global health environments, where a CPR using only three predictors may be particularly useful. Furthermore, an analysis of routinely collected foot risk assessment data conducted by the wider research group found only 5% of people at low risk of foot ulceration changed their risk score during a 2-year period.^36^ This suggests that biennial, as opposed to annual foot risk assessment for those at low risk of foot ulceration may be adequate.^36^ If this simplified CPR was used biennially to assess risk of foot ulceration, the burden on diabetes services across primary care, community and hospital settings could be greatly reduced.

CPRs are a form of Clinical Decision Support System (CDSS), but these can be underused unless they are integrated into existing clinical systems. For example, CDSSs requiring computing software need to be embedded into the IT systems and the healthcare professionals are obliged to use in routine care.^37 38^ A CPR that can be used independently of electronic equipment is of value because of the very wide range of IT availability for healthcare professionals across all clinical settings worldwide. In this manuscript, there is a print version of the CPR for use in the consulting room (online supplemental material), and any healthcare professional could learn the risk estimates for each individual score quickly. However, the CPR could also be integrated into existing electronic foot screening programs such as SCI-diabetes (https://www.sci-diabetes.scot.nhs.uk/).

The CPR was developed using individual patient data from three different countries and healthcare systems to make a better assessment of the generalizability and applicability across different healthcare settings and populations.^39^ The authors are unaware of any other CPRs for foot ulceration in diabetes that are equally simple to use and that have had such a robust process of development and validation.

The four international cohort studies which were used in the analyses were designed specifically to identify predictive factors of foot ulceration in people with diabetes. These structured datasets had very few missing values, a characteristic of routinely collected datasets which can undermine the process of validation for prognostic models.^40^

### Strengths and limitations

Despite being derived from only three binary predictors, the discrimination and calibration of the CPR were excellent when externally validated in a UK population. There was slight miscalibration in those with the highest risks, but this is acceptable if clinical risk thresholds are likely to be much lower. Indeed, the net benefit analysis suggests that the use of the CPR to identify people who score 1 or above (ie, predicted risk of 6% or above) for subsequent treatment with preventative interventions could bring greater clinical benefits than either treat-all or treat-none strategies and thus is likely to have clinical utility in practice. Further validation in countries other than the UK, Portugal, or the USA would be welcome.

The weaknesses of the CPR and the underlying statistical model lie in its simplicity; as it has only three binary predictors, it will not give predictions across the entire 0 to 1 probability range. However, there are no prognostic models that predict foot ulceration with 100% accuracy and all comprise more sophisticated and expensive tests than this CPR. Also, many outcomes in diabetes are dependent on self-care, and in particular, the maintenance of a tight HbA1c and a body mass index (BMI) lower than 27. Individuals can exert some control over these parameters. HbA1c has a known association with the development of peripheral neuropathy (the main etiology for foot ulceration in diabetes).^41^ Initially, HbA1c and BMI were considered for the model in PODUS 2015; however, neither were collected in the largest study.

As CPRs are not treatments in themselves, they do not directly influence clinical outcomes unless they are linked to clinical decision thresholds. The net benefit analysis shows that the CPR has the potential to have clinical utility when a score of one or above is used to trigger treatment, but further evaluation is required where preventative interventions are targeted at those with a score of one or more. As death has also been linked to foot ulceration, ideally, an impact study would account for those participants who died before the end of the 2 years’ follow-up and would analyze time-to-ulceration events and incorporate a competing risk model.

The use of this CPR in conjunction with effective preventative interventions could improve patient outcomes by reducing the number of foot ulcers and generate financial savings for the NHS. The simplicity of the CPR means that the cost implications of implementing it in clinical practice are minimal.

The CPR was developed and validated in a large international dataset, but an evaluation of its clinical impact in different patient populations would assess its therapeutic impact. This should be assessed in a prospective comparative study, preferably a randomized controlled trial.

## Data Availability

Data are available on reasonable request. All requests for data must be made in the first instance to FC.
