# Supplementary material for: Development and validation of a clinical prediction rule for development of diabetic foot ulceration: an analysis of data from five cohort studies
Source: BMJ Open Diabetes Res Care. 2021 May 25;9(1):e002150. doi: 10.1136/bmjdrc-2021-002150 (PMC8154962; doi:10.1136/bmjdrc-2021-002150)
Supplement: Supplementary data [file bmjdrc-2021-002150supp001.pdf]

ONLINE SUPPLEMENT

Background to PODUS 2020

This PODUS 2020 project is a continuation of the work started in the PODUS 2015 project. The results of the PODUS 2015 project have been published as part of the NIHR HTA monograph series (https://doi.org/10.3310/hta19570).

Studies were identified via systematic review and the authors of eight studies[1-8] agreed to give their data. A ninth study was made available via a Safe Haven facility,[9, 10] and the author of a tenth study could not make the data available, but SAS programs (www.sas.com) were supplied to the study statistician so that she could run analyses and return the results to the PODUS team [11].

Selection of predictors

For PODUS 2015, a list of the potential predictors of foot ulceration was made based on clinical plausibility and availability in the datasets. These predictors were:

Age
Sex
Body mass index
Smoking
Height
Weight
Alcohol use
HbA1c

Insulin regime
Duration of diabetes
Eye problems
Kidney problems
Insensitivity to a 10g monofilament
Absent pedal pulses
Tuning fork

Biothesiometer
Ankle reflexes
Ankle-brachial index
Peak plantar pressure
Previous ulcer
Previous amputation
Foot deformity

These predictors were discussed at an investigators' meeting to choose which should be included in the final model. Several were rejected at this stage for inconsistency of definition across the studies. For example, "eye problems" ranged from overt retinopathy to wearing glasses. Other predictors had been collected in only one or two studies and so were also rejected at this stage. The final PODUS 2015 model had six predictors:

Age
Sex

Duration of diabetes
Monofilaments

Absent pedal pulses
Previous ulcer/amputation

The PODUS 2015 outcome variable was development of a new foot ulcer at any time during the study follow-up. The statistician of the tenth study tested these six predictors using SAS programs supplied by the PODUS team, and she was not otherwise involved in PODUS.

Results were considered to be replicated if the results in the tenth study coincided with those from the eight PODUS studies. The coefficient for each predictor had to be in the same direction in both the PODUS analyses and those from the tenth study, the confidence intervals had to overlap, and the predictor had to achieve statistical significance in the tenth dataset. This process replicated three predictors:

Monofilaments

Absent pedal pulses

Previous ulcer/amputation

Age was not a statistically significant predictor in either the PODUS analyses or in the tenth study. Increasing duration of diabetes increased the odds of foot ulcer in our analyses, but decreased the odds in the tenth dataset. Non-linearity in relationships was checked as an explanation of these results, but no evidence was found of any non-linearity.[12] It turned out that the tenth dataset had very few women (<2%) and so could not be used to confirm sex as a predictor.

Sample size

There was no sample size calculation as the datasets were pre-existing and the analysis simply used all the data available. However, recent sample size guidance [13, 14] was checked, and the size of the dataset and number of outcomes were adequate for the analyses.

The development datasets had 8255 participants, who had 430 ulcer outcomes, which gave 143 events per predictor parameter. Assuming a conservative model performance (Cox-Snell R^2 of 15%), this exceeded the recommended minimum sample size for model development.[14]

In the validation dataset, 295 participants were removed from the analysis as they had already contributed data to one of the development datasets.[2] This reduced the validation dataset from 3707 to 3412. The validation dataset had 128 ulcer outcomes, again exceeding the recommendation of at least 100 events and 100 non-events to validate model performance in an external dataset.[15]

Missing data

The percentage of participants that had missing data for predictors or outcome was calculated. Some participants were missing data on previous history of ulceration or amputation. As both ulceration and amputation are important to record, it was judged that missing data on these events meant that the participant had no previous history of ulceration or amputation. Therefore missing data for history was recoded as negative for this predictor. For the other predictors, other methods for dealing with missing data, such as multiple imputation, would have been considered, but as the proportion of missing data in the development studies was <2.4% and in the validation dataset <2.6%, a complete-case analysis was performed.

Participant flowcharts for each study (see supplementary material) show where data were missing and the effect of recoding of missing data for previous history.

Handling competing risk of death

Some participants died in each study before the end of follow-up. In the community-based studies, one death was recorded in the largest dataset over its two year follow-up period,[1] and 59 people died in the dataset with one year follow-up.[2] In the two secondary care studies, one recorded that 13 people had died,[6] but death was not recorded in the other study.[5] Since death was not systematically recorded in all studies, participants were included whether or not it was known they had died, provided they had complete data on the predictors and outcome before their death. The total number of known deaths (73) comprised less than 1% of the analysis dataset. The CPR therefore assumes that people who died before developing a foot ulcer would not have a foot ulcer by two years.

During the two-year follow-up period of the validation dataset, 95 patients died, 2.8% of 3412. We applied the same method as to the development datasets, and included these people in the analyses if they had complete data on predictors and outcome.

Statistical analysis plan

The analysis used a logistic regression model with random effects on the intercept, so that each study could have a different baseline risk of ulcer by two years. However, one of the development studies only had follow-up for one year, not two[2]. This study did not contribute to the overall estimate of baseline risk in the prognostic model, but it was allowed to contribute to the estimates of odds ratios (see supplementary material), which were deemed similar enough at one year or two years to combine (and preferable to simply excluding the study and losing a large number of participants).

After model development, the potential for overfitting was estimated by calculating a heuristic shrinkage factor. Shrinkage estimates close to one suggest that the model's estimates are not optimistic (i.e. overfitting is of little concern), whereas smaller shrinkage estimates suggest that the model's predictions are optimistic and should be shrunk.

When calculating risks for each score of the CPR, population-averaged risk estimates were calculated, which use the random effects distribution of baseline risks rather than one summary estimate of baseline risk, to allow for the data being clustered in studies. Population-averaged estimates are considered to be more generalizable to participants in new studies.[16]

Steyerberg's method for developing a clinical prediction rule from a statistical model was used [17]. However, the step where the coefficients of predictors are made smaller to compensate for overfitting was omitted. Overfitting is a named given to the phenomenon where statistical models tend to perform better in the datasets they were derived from than independent datasets. This is a particular problem for small datasets, complex models, or large numbers of predictors. The CPR dataset was large, the model simple, and the number of predictors small, and the extent of overfitting with shrinkage factors was estimated and found it to be negligible. Shrinkage was >0.999 in all cases.

Shrinkage was estimated by: [18]

$$\frac{Likelihood\ ratio\ \chi^2 - number\ of\ predictor\ parameters}{Likelihood\ ratio\ \chi^2}$$

Where overfitting occurs, it is recommended that the coefficients of the model are adjusted by multiplying by the shrinkage factor.

As the development dataset comes from four studies, this was accounted for in the analysis by allowing the individual studies to have different baseline risk of ulcer by two years. However, in one of the studies the length of follow-up was only one year [2], Therefore, the baseline risk of ulcer in this study was lower than in the other studies as the participants had less time to develop an ulcer. To address this, the study's Principal Investigator attempted to obtain longer-term follow-up data with limited success, and the PODUS 2020 steering committee advised not to use the longer-term follow-up data.

The prediction model's baseline risk of ulcer at two years was estimated with data from the three studies with at least two-year follow-up [1, 5, 6]. First, a logistic regression model was fitted with study as a predictor in addition to the three clinical predictors to obtain baseline risk estimates for each study. Then a random effects meta-analysis of the three study-specific baseline risk estimates was conducted to obtain an overall baseline risk estimate for the prediction model, giving an estimated risk at two years conditional on the three clinical predictors. The estimates for the three

clinical predictors used all four studies. Heterogeneity in the effect of the clinical predictors was not modelled.

The implementation of Steyerberg's method was:

1. Fit the logistic regression model with monofilament, pulses, previous history of ulcer/amputation, and study as predictors. This gives coefficients showing by how much the log odds changes when monofilaments, pulses, or history change from test-negative to test-positive and estimates of baseline risk for each study. The software used was SAS PROC LOGISTIC (SAS 9.4 www.sas.com) with maximum likelihood estimation.
2. Perform a random-effects meta-analysis of the three estimates from the studies with two years of follow-up to get a single overall estimate of baseline risk.
3. Use this overall estimate and the regression coefficients for the three predictors to calculate the probability of ulcer for each possible predictor combination. There are three binary predictors and therefore eight possible predictor combinations.
4. Multiply and round the coefficients of the predictors to get a CPR scoring scheme, bearing in mind that predictor combinations with similar risk of ulcer should have the same score.
5. Repeat Step 1 and Step 2, only using the CPR score instead of monofilaments, pulses, and history.
6. Calculate probability of ulcer for each score using a population average method.[16] The population average method should produce estimates with better calibration in external datasets and generalisability to people recruited to new studies than simply using the CPR logistic regression equation.

Risk of bias assessment with PROBAST tool

Table 1 Results of PROBAST[19] evaluation for the development studies (Abbott, Crawford, Monteiro-Soares, Pham) and validation study (Leese)

Study	Risk of bias			Applicability			Overall	
	Participants	Predictors	Outcome	Participants	Predictors	Outcome	Risk of bias	Applicability
Abbott	+	+	+	+	+	+	+	+
Crawford	+	+	+	+	+	+	+	+
Monteiro-Soares	+	+	+	+	+	+	+	+
Pham	+	+	+	+	+	+	+	+
Leese	+	+	-	+	+	+	-	+

Flowcharts of participants for each study.

Note that a flowchart for the Monteiro-Soares study was omitted as there were no missing data.

Figure S1 Flow of patients in Abbott dataset. All patients had two year ulcer outcome recorded. Not all patients are shown at each stage.

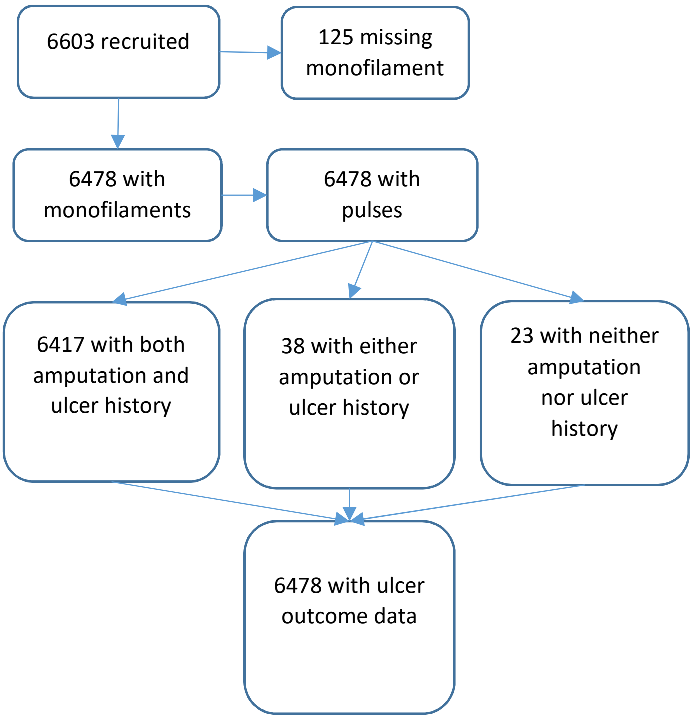

Figure S2 Flow of patients in the Crawford dataset. Not all patients are shown at each stage.

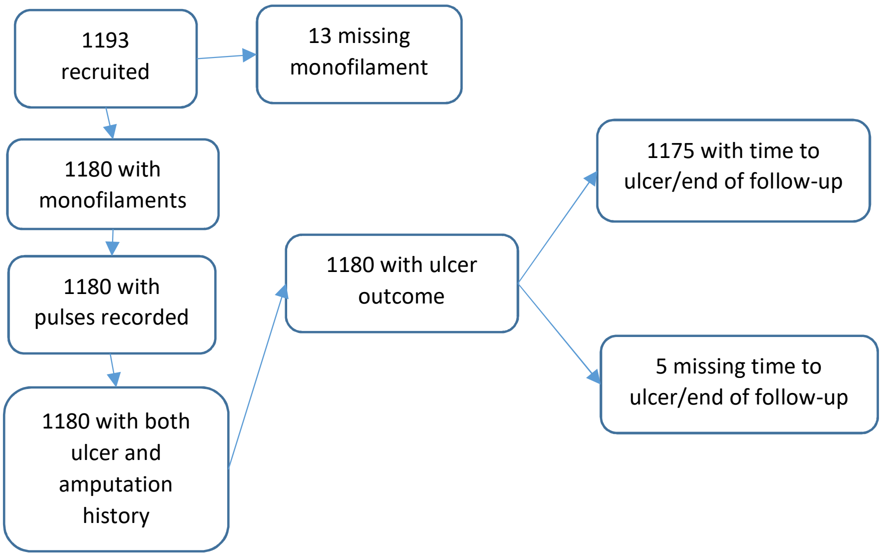

Figure S3 Flow of patients in the Pham study. Not all patients are shown at each stage.

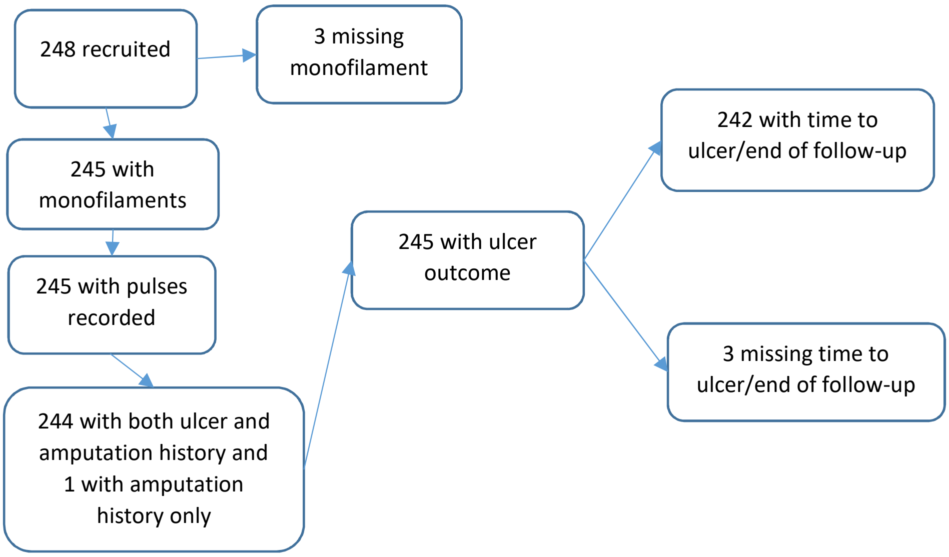

Figure S4 Flow of patients in the Leese study. Not all patients are shown at each stage.

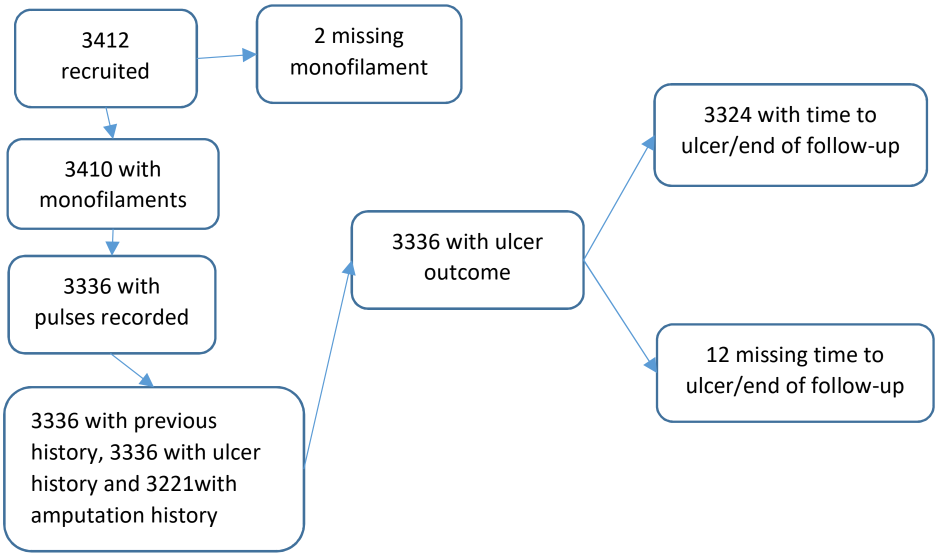

Development of the CPR from the prognostic logistic regression model

Overfitting in the logistic regression model was negligible, with shrinkage estimated as > 0.999, so there was no need to adjust coefficients.

On the log odds scale, the initial logistic regression model with original predictors (coded 0 if test-negative and 1 if test positive so that a 1 always indicated "disease") was:

$$Log\ odds\ of\ ulcer\ by\ two\ years = -3.81 + 1.11 * mono + 0.70 * pulse + 1.95 * history$$

The intercept of -3.81 came from the random effects meta-analysis of study-specific baseline risk of the three studies with two-year follow-up data. Based on this model, predicted risks are:

$$Risk\ of\ ulcer\ at\ two\ years = \frac{1}{1 + e^{-(-3.81+1.11*mono+\ .70*pulse\quad .95*history)}}$$

Repeating the analysis with CPR score gave this equation:

$$Risk\ of\ ulcer\ at\ two\ years = \frac{1}{1 + e^{-(-3.73+0.944*score)}}$$

Again, overfitting for the model with CPR score as assessed by the shrinkage factor (>0.999) was negligible. This equation was not used directly to calculate the risk of ulcer, but instead the population averaged method of Pavlou et al.[16] Here both approaches give similar results. The Pavlou method uses the distribution of random effects rather than just the point estimate of -3.73 when estimating the risks. The risk of ulceration for each score is given in Table 3 of the manuscript.

Calibration in the validation dataset of the prognostic model and CPR score

Table 2 External data calibration statistics for the three predictor and CPR models.

Model	Calibration-in-the-large (95% CI)	Calibration slope (95% CI)
Full prognostic model	-0.269 (-0.457 to -0.082)	1.133 (0.990 to 1.276)
CPR score	-0.374 (-0.561 to -0.187)	1.139 (0.994 to 1.283)

Discrimination in the validation dataset of the prognostic model and CPR score.

Figure S5 External validation ROC plot from the validation dataset for both the prognostic model with three predictors and the CPR score.

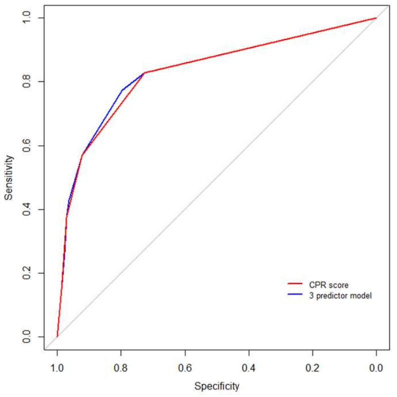

Net benefit

The potential clinical utility of the CPR was assessed with a net benefit analysis. At a risk threshold of 6% the net benefit is 0 for treat none, and < 0 for treat all, but 0.015 for using the CPR. This can be interpreted as the decision was to treat patients with CPR scores of 1 and above, then 15 additional cases of ulcer at 2 years would be correctly identified for treatment by the CPR, without increasing the number treated unnecessarily, per 1000 individuals. At a risk threshold of 14%, the number of additional cases of ulcer at 2 years correctly identified for treatment would be 10 per 1000 individuals. See decision curves in Figure S6.

Figure S6 Net benefit plot with decision curves for “treat none”, “treat all”, and “treat according to CPR score”.

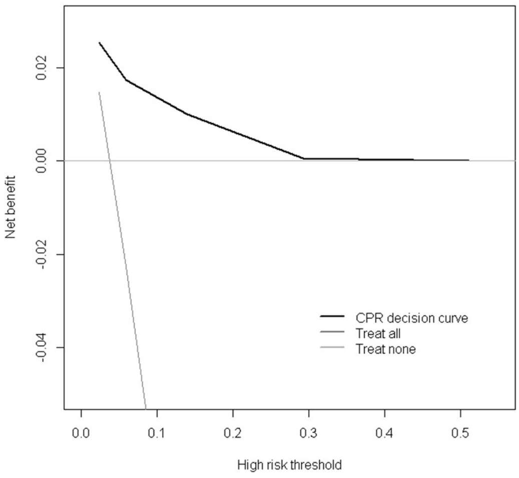

Printable CPR

PODUS Clinical Prediction Rule	Score
Test with 10g monofilament<br><br>Insensitive at any site(s) – score 1 point<br>Sensitive at all sites – score 0 points	
Check pedal pulses<br><br>Any pulse(s) missing – score 1 point<br>4 pulses present – score 0 points	
Has there been an ulcer or amputation previously?<br><br>Any ulcer or amputation – score 2 points<br>No ulcer or amputation – score 0 points	
Total score out of 4	

Score	Risk of ulcer at two years
0	2%
1	6%
2	14%
3	29%
4	51%

REFERENCES

[1] Abbott CA, Carrington AL, Ashe H, et al. (2002) The North-West Diabetes Foot Care Study: incidence of, and risk factors for, new diabetic foot ulceration in a community-based patient cohort. Diabet Med 19(5): 377-384

[2] Crawford F, McCowan C, Dimitrov BD, et al. (2011) The risk of foot ulceration in people with diabetes screened in community settings: findings from a cohort study. Q J Med 104(5): 403-410. 10.1093/qjmed/hcq227

[3] Kästenbauer T, Sauseng S, Sokol G, Auinger M, Irsigler K (2001) A prospective study of predictors for foot ulceration in Type 2 diabetes. J Am Podiatr Med Assoc 91(7): 343-350. 10.7547/87507315-91-7-343

[4] Monami M, Vivarelli M, Desideri CM, Colombi C, Marchionni N, Mannucci E (2009) Pulse pressure and prediction of incident foot ulcers in Type 2 diabetes. Diabetes Care 32(5): 897-899

[5] Monteiro-Soares M, Dinis-Ribeiro M (2010) External validation and optimisation of a model for predicting foot ulcers in patients with diabetes. Diabetologia 53(7): 1525-1533. 10.1007/s00125-010-1731-y

[6] Pham H, Armstrong DG, Harvery C, Harkless LB, Giurini JM, Veves A (2000) Screening techniques to identify people at high risk for diabetic foot ulceration. Diabetes Care 23(5): 606-611

[7] Rith-Najarian SJ, Stolusky T, Gohdes DM (1992) Identifying Diabetic Patients at High Risk for Lower-Extremity Amputation in a Primary Health Care Setting: A prospective evaluation of simple screening criteria. Diabetes Care 15(10): 1386-1389. 10.2337/diacare.15.10.1386

[8] Young MJ, Breddy JL, Veves A, Boulton AJM (1994) The Prediction of Diabetic Neuropathic Foot Ulceration Using Vibration Perception Thresholds: A prospective study. Diabetes Care 17(6): 557-560. 10.2337/diacare.17.6.557

[9] Leese GP, Cochrane L, Mackie ADR, Stang D, Brown K, Green V (2011) Measuring the accuracy of different ways to identify the 'at-risk' foot in routine clinical practice. Diabet Med 28(6): 747-754. 10.1111/j.1464-5491.2011.03297.x

[10] Leese GP, Reid F, Green V, et al. (2006) Stratification of foot ulcer risk in patients with diabetes: a population-based study. Internation Journal of Clinical Practice 60(5): 541-545. 10.1111/j.1368-5031.2006.00899.x

[11] Boyko EJ, Ahroni JH, Cohen V, Nelson KM, Heagerty PJ (2006) Prediction of Diabetic Foot Ulcer Occurrence Using Commonly Available Clinical Information: The Seattle Diabetic Foot Study. Diabetes Care 29(6): 1202-1207. 10.2337/dc05-2031

[12] Osbourne JW (2015) Curvilinear Effects in Logistic Regression. In: Best Practices in Logistic Regression. SAGE Publications Ltd, London, pp 201-242

[13] Goodman SN, Berlin JA (1994) The use of predicted confidence intervals when planning experiments and the misuse of power when interpreting results. Ann Intern Med 121(3): 200-206

[14] Riley RD, Ensor J, Snell KIE, et al. (2020) Calculating the sample size required for developing a clinical prediction model. BMJ 368. 10.1136/bmj.m441

[15] Collins GS, Ogundimu EO, Altman DG (2016) Sample size considerations for the external validation of a multivariable prognostic model: a resampling study. Stat Med 35(2): 214-226. 10.1002/sim.6787

[16] Pavlou M, Ambler G, Seaman S, Omar RZ (2015) A note on obtaining correct marginal predictions from a random intercepts model for binary outcomes. BMC Med Res Methodol 15. 10.1186/s12874-015-0046-6

[17] Steyerberg EW (2009) Clinical Prediction Models: a Practical Approach to Development, Validation, and Updating. Springer, New York

[18] van Houwelingen JC, Le Cessie S (1990) Predictive value of statistical models. Stat Med 9(11): 1303-1325

[19] Moons KGM, Wolff RF, Riley RD, et al. (2019) PROBAST: A Tool to Assess Risk of Bias and Applicability of Prediction Model Studies: Explanation and Elaboration. Ann Intern Med 170(1): W1-W33. 10.7326/M18-1377
